# Rh-CXCL-12 Attenuates Neuronal Pyroptosis after Subarachnoid Hemorrhage in Rats via Regulating the CXCR4/NLRP1 Pathway

**DOI:** 10.1155/2021/6966394

**Published:** 2021-11-09

**Authors:** Ran Gu, Lu Wang, Hao Zhou, Xike Wang, Cameron Lenahan, Hao Qu, Yonghe Liu, Shirong Li, Changxiu Wei, Lu Han, Xiao Hu, Gang Zuo

**Affiliations:** ^1^Department of Neurology, Guizhou Provincial People's Hospital, Guiyang 550002, China; ^2^Department of Pediatric, Guizhou Provincial People's Hospital, Guiyang 550002, China; ^3^Burrell College of Osteopathic Medicine, Las Cruces, NM 88003, USA; ^4^Department of Clinical Laboratory, Guizhou Provincial People's Hospital, Guiyang 550002, China; ^5^Department of Neurosurgery, Taicang Hospital Affiliated to Soochow University, Taicang, Suzhou, Jiangsu 215400, China

## Abstract

Subarachnoid hemorrhage (SAH) is a cerebrovascular disease associated with high morbidity and mortality. CXCR4 provides neuroprotective effects, which can alleviate brain injury and inflammation induced by stroke. Previous studies have suggested that CXCR4 reduces the pyroptosis of LPS-stimulated BV2 cells. The purpose of this study was to evaluate the antipyroptosis effects and mechanisms of CXCR4 after SAH. SAH animal model was induced via endovascular perforation. A total of 136 male Sprague-Dawley rats were used. Recombinant human cysteine-X-cysteine chemokine ligand 12 (rh-CXCL-12) was administered intranasally at 1 h after SAH induction. To investigate the underlying mechanism, the inhibitor of CXCR4, AMD3100, was administered intraperitoneally at 1 h before SAH. The neurobehavior tests were assessed, followed by performing Western blot and immunofluorescence staining. The Western blot results suggested that the expressions of endogenous CXCL-12, CXCR4, and NLRP1 were increased and peaked at 24 h following SAH. Immunofluorescence staining showed that CXCR4 was expressed on neurons, microglia, and astrocytes. Rh-CXCL-12 treatment improved the neurological deficits and reduced the number of FJC-positive cells, IL-18-positive neurons, and cleaved caspase-1(CC-1)-positive neurons after SAH. Meanwhile, rh-CXCL-12 treatment increased the levels of CXCL-12 and CXCR4, and reduced the levels of NLRP1, IL-18, IL-1*β*, and CC-1. Moreover, the administration of AMD3100 abolished antipyroptosis effects of CXCL-12 and its regulation of CXCR4 post-SAH. The CXCR4/NLRP1 signaling pathway may be involved in CXCL-12-mediated neuronal pyroptosis after SAH. Early administration of CXCL-12 may be a preventive and therapeutic strategy against brain injury after SAH.

## 1. Background

The novel coronavirus disease 2019 (COVID-19) is a global pandemic with international concern. Aneurysmal subarachnoid hemorrhage (SAH) is a devastating and life-threatening disease associated with high mortality and disability [1, 2]. The annual worldwide incidence of SAH is approximately 9.1 per 100,000 people [3], and SAH resulting from intracranial aneurysm rupture accounts for 5-10% of strokes [4]. There is an association between the SAH and COVID-19 infection [5]. Early brain injury (EBI) appears in the first 3 days following SAH, which is the major cause of poor prognosis regarding high mortality and delayed neurological deficits.

EBI begins immediately after a ruptured intracranial aneurysm, which has been proven to be the primary cause of poor outcome after SAH. During the EBI period, ruptured intracranial aneurysms result in many physiological derangements, including elevated intracranial pressure, decreased cerebral blood flow, and global cerebral ischemia [6]; all of which initiate a variety of pathophysiological events, such as oxidative stress, neuroinflammation, blood–brain barrier dysfunction, apoptosis, and pyroptosis [7, 8]. Pyroptosis induced by SAH has been considered a critical devastating pathophysiological process in EBI after SAH [9].

Inflammasome is a multiprotein complex, which is a significant factor in the mechanism of pyroptosis [10]. As the first member of the NACHT Leucine-rich (NLR) family, NACHT Leucine-rich-repeat protein 1 (NLRP1) is mainly expressed on neurons and microglia [11]. After the assembly of NLRP1 inflammasome, cleaved caspase-1 (CC-1) is activated, and pro-IL-1 *β* and pro-IL-18 are converted into IL-1*β* and IL-18, which regulate pyroptosis. Previous studies have found that CC-1 was highly expressed in the wall of human brain aneurysms and was overexpressed in ruptured aneurysm tissue, indicating that pyroptosis was involved in the occurrence of SAH [12]. Other studies have found that the levels of NLRP1, ASC, and CC-1 in the cerebrospinal fluid of SAH patients are higher than the control group, which were associated with poor prognosis. Therefore, high level of NLRP1 is an independent risk factor for unfavourable prognosis after SAH [13].

Cysteine-X-cysteine chemokine ligand 12 (CXCL-12) is an inflammatory chemokine derived from bone marrow mesenchymal stem cells and belongs to the CXC chemokine family. CXC chemokine receptor type 4 (CXCR4) is one of the seven transmembrane G-protein-coupled receptors that mediates the transmembrane signaling of CXCL-12. CXCL-12 and CXCR4 are abundant and widely expressed in the central nerves system (CNS), playing important roles in neurogenesis and contributing to the neuronal development [14]. CXCL-12 is known to be expressed in neurons, glial cells, endothelial cells, and meningeal cells [15]. Previous studies have shown that CXCL-12 is upregulated in the penumbra after stroke, reduces neurological deficits, and promotes angiogenesis [16–18]. CXCR4 is reportedly expressed in neurons, astrocytes, microglia, and ependymal cells [19]. Recent studies have demonstrated that CXCR4 is involved in the inflammatory response and exerts neuroprotective effects after ischemic stroke [20, 21]. Roosen et al. have suggested that CXCR4 reduces the pyroptosis of LPS-stimulated BV2 cells [22]. Other studies have reported that CXCL-12 significantly reduces the levels of IL-18, IL-1*β*, TNF-a, NLRP3, ASC, and CC-1 in a spinal cord injury model [23]. However, no research has been investigated to explore the role of CXCR4 and the underlying mechanism after SAH.

**Figure 1:**
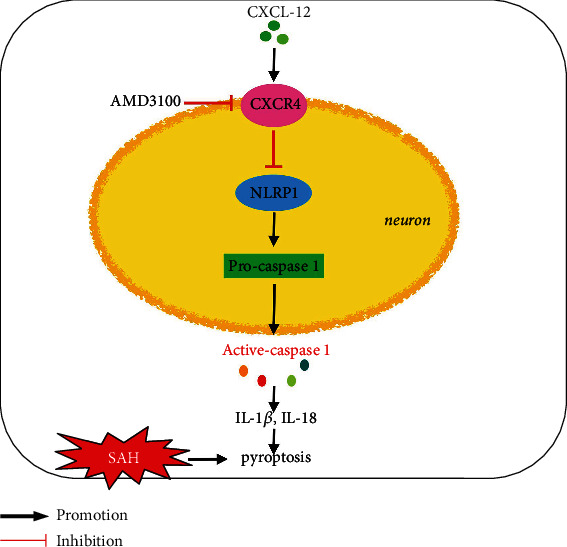
Schematic summary. Rh-CXCL-12 attenuates neuronal pyroptosis via the CXCR4/NLRP1 signaling pathway in a rat model of SAH.

In the present study, we hypothesized that rh-CXCL-12 would attenuate neuronal prognosis by inhibiting NLRP1 and improve neurological impairment. Moreover, these beneficial effects were at least in part via regulation of CXCR4/NLRP1 signaling pathway after SAH in rats (Figure 1).

## 2. Materials and Methods

### 2.1. Animals

Adult male Sprague-Dawley rats (weighting 280-330 g, *n* = 136) obtained from Guizhou Laboratory Animal Engineering Technology Center (China) were used in this project. All animals were kept in a room with controlled humidity (60 ± 5%) and constant temperature (25 ± 1°C), and remained in a 12 h light and dark cycle and with ad libitum access to food and water.

### 2.2. SAH Model

The SAH model was performed in rats using a modified endovascular perforation model as previously described [24]. Induction of anesthesia in rats was achieved using 4% isoflurane and was maintained using 2.5% isoflurane. After intubation, the mice were placed in the supine position and connected to the rodent ventilator to breathe medical air (70%) and oxygen (30%). The heart rate, respiration, skin color, and pedal reflex assessment were assessed every five minutes during the operation to confirm anesthesia status and prevent distress. After exposing the carotid artery and its bifurcation, a 4-0 sharp single nylon thread suture was inserted from the external carotid artery into the left internal carotid artery to the anterior and middle cerebral artery bifurcation. The nylon suture was withdrawn immediately, and isoflurane was reduced to 1.5%. After the operation, the endotracheal tube was removed and the animals were placed in the heating chamber (37.5°C) to recover. Animals in the sham group underwent the same procedure, but without arterial wall puncture.

### 2.3. SAH Grading

The degree of SAH was assessed according to the SAH grading scale system at 24 h after SAH as previously described [25]. Rats with a score of 8 or less were excluded from the current study.

### 2.4. Experimental Design

Four separate experiments were performed as follows.

#### 2.4.1. Experiment 1

To determine the time course of endogenous CXCL-12, CXCR4, and NLRP1 protein level expression in the sham group and each group after SAH. The rats were randomly divided into six groups (*n* = 6/group): sham, SAH-6 h, SAH-12 h, SAH-24 h, SAH-48 h, and SAH-72 h. Western blot was performed to assess the protein levels of CXCL-12, CXCR4, and NLRP1 in the ipsilateral (left) hemisphere cerebral cortex. Additionally, the cellular localization of CXCR4 with calcium-binding adaptor molecule 1 (Iba-1), *neuronal nucleus* antigen (NeuN), and glial fibrillary acidic protein (GFAP) was evaluated using double immunofluorescence staining in the sham and SAH-24 h group (*n* = 2/group).

#### 2.4.2. Experiment 2

To evaluate the neuroprotective effects of CXCL-12 on short-term neurological outcomes after SAH, rats were randomly assigned to five groups (*n* = 6/group): sham, SAH+vehicle (sterile distilled water), SAH+CXCL-12 (5 *μ*g/kg), SAH+CXCL-12 (15 *μ*g/kg), and SAH+CXCL-12 (45 *μ*g/kg). CXCL-12 was administered intranasally (i.n.) at 1 h after SAH. The SAH grading score, neurobehavioral test (including modified Garcia test and beam balance test), and brain water content were assessed at 24 h after SAH in all groups. The best dose of CXCL-12 was selected based on the short-term neurological outcomes and brain water content results, which was also used for the mechanism experiments.

#### 2.4.3. Experiment 3

To explore the effects of CXCL-12 on neuronal degeneration and pyroptosis at 24 h after SAH, rats were randomly assigned to three groups (*n* = 4/group): sham, SAH+vehicle (sterile distilled water), and SAH+CXCL-12 (optimal dose). Fluoro-Jade C (FJC) staining was performed, and double immunofluorescence staining was used to evaluate the CC-1 and IL-18 expression on neurons in the perihemorrhagic area at 24 h after SAH. Numbers of FJC-positive cells, CC-1-positive neurons, and IL-18-positive neurons were counted. Brain samples of these three groups were shared with experiment 4.

#### 2.4.4. Experiment 4

To explore the underlying mechanism of the CXCR4/NLRP1 signaling pathway-mediated antipyroptosis effects after SAH, the selective CXCR4 inhibitor, AMD3100, was administered intraperitoneally (i.p.) at 1 h before SAH. Rats were randomly assigned to five groups (*n* = 6/group): sham, SAH+vehicle (sterile distilled water, i.n.), SAH+CXCL-12, SAH+CXCL-12+AMD3100, and SAH+CXCL-12+PBS (vehicle of AMD3100). The ipsilateral (left) hemisphere of each group was collected for Western blot analysis (*n* = 6/group) after neurological performance, and SAH grades were evaluated at 24 h after SAH.

### 2.5. Drug Administration

CXCL-12 or vehicle was given via intranasal administration at 1 h after SAH as previously described [26]. Animals were placed in the supine position and were administered 1.5% isoflurane anesthesia. A total volume of 20 *μ*L of vehicle (sterile distilled water) or CXCL-12 (MedChem Express, NJ, USA) at three different doses (5 *μ*g/kg, 15 *μ*g/kg, and 45 *μ*g/kg), with 5 *μ*L administered every 5 minutes, alternating between the right and left nares. AMD3100 was diluted in PBS and administered intraperitoneally (i.p.) at 1 h before SAH.

### 2.6. Assessment of Short-Term Neurological Performance

The short-term neurobehavioral outcomes were assessed blindly using the 18 points modified Garcia scoring system and the 4 points beam balance test at 24 h after SAH as previously described [27]. Higher scores indicated better neurological function.

### 2.7. Brain Water Content

Brain edema was assessed by measuring brain water content using the wet-dry method as previously described [8]. The rats were euthanized at 24 h after SAH, and the brains were quickly removed and separated into four parts (right hemisphere, left hemisphere, cerebellum, and brain stem). Afterwards, each part of the brain was weighed immediately to obtain the wet weight and then placed into an oven for 72 h at 100°C. The dried brain was weighed again. The percentage of brain water content was calculated as follows: (wet weight − dry weight)/wet weight × 100%.

### 2.8. Immunofluorescence Staining

The rats were deeply anesthetized (5% isoflurane) and euthanized via transcardiac perfusion with 100-150 mL of precooled PBS (4°C) and 100 mL of 10% formalin. Whole brains were rapidly collected and fixed in 10% formalin (4°C, 24 h), followed by dehydration with 30% sucrose (4°C, 72 h). Brain samples were embedded in OCT (Scigen Scientific, Gardena, CA, USA), and then frozen at −80°C. The brains were sliced into 10 *μ*m thick coronal brain sections using a cryostat (CM3050S, Leica Microsystems, Bannockburn, Germany) and then mounted onto normal poly-L-Lysine-coated slides. The slices were washed with 0.01 M of PBS three times for 5-10 min and then incubated in 0.3% Triton X-100 in 0.01 M of PBS for 10 min at room temperature. After being blocked with 5% donkey serum in 0.01 M of PBS for 2 h at room temperature, the sections were incubated overnight at 4°C with the following primary antibodies: anti-Iba-1 (1 : 200, Abcam), anti-NeuN (1 : 200, Abcam), anti-GFAP (1 : 200, Abcam), anti-CC-1 (1 : 100, Santa Cruz Biotechnology), anti-IL-18 (1 : 200, Abcam), and anti-CXCR4 (1 : 200, Abcam). Next, the slices were incubated with fluorescence-conjugated secondary antibodies (1 : 500) for 1 h at room temperature. The slides were visualized and photographed using a fluorescence microscope. To assess neuronal pyroptosis levels, the number of CC-1-positive neurons and IL-18-positive neurons was identified and counted in three different fields from the left basal cortex of five random coronal sections of each rat. The positive cells were quantified under a microscopic field of 200x magnification, and data were expressed as cells/field.

### 2.9. FJC Staining

To detect neuronal degeneration, FJC staining was performed using the FJC Ready-to-Dilute Staining Kit (Biosensis, USA) according to manufacturer's protocol. The stained slices were observed and photographed under a fluorescence microscope and analyzed by Leica Application Suite software. The brains of four rats per group were counted from 5 fields per brain in the per lesion region for quantification analysis. The FJC-positive cells were quantified under a microscopic field of 200x magnification, and data were expressed as cells/field.

### 2.10. Western Blot Analysis

At 24 h after SAH, rats were deeply anesthetized (5% isoflurane) and transcardially perfused with chilled PBS, followed by decapitation. The brain sections were separated into ipsilateral and contralateral hemispheres. The ipsilateral hemisphere brain tissues were snap frozen in liquid nitrogen and stored in a −80°C freezer for storage until used. Brain samples were homogenized in RIPA lysis buffer with protease inhibitor for 15 min and then centrifuged at 14,000 g (4°C, 30 min). The supernatant was collected, and protein concentration was measured by detergent compatible assay (DC Protein Assay, Bio-Rad Laboratories). Equal amounts of protein were loaded onto the 10% SDS-PAGE gel for electrophoresis and then transferred onto nitrocellulose membranes. The membranes were blocked with 5% nonfat blocking grade milk for 2 h (37°C) and incubated overnight at 4°C with the following primary antibodies: anti-CXCR4 (1 : 1000, Abcam), anti-CXCL-12 (1 : 1000, Abcam), anti-NLRP1 (1 : 1000, Abcam), anti-CC-1 (1 : 1000, Cell Signaling Technology), anti-IL-18 (1 : 1000, Abcam), anti-IL-1*β* (1 : 1000, Abcam), and anti-*β*-actin (1 : 5000, Santa Cruz Biotechnology). The membranes were incubated with the appropriate peroxidase-conjugated secondary antibodies (1 : 5000, Santa Cruz) for 1 h at 37°C. The bands were then visualized with the ECL Plus chemiluminescence reagent kit (Amersham Bioscience, Pittsburgh, PA) and quantified with the ImageJ software (ImageJ 1.5, NIH, USA).

### 2.11. Statistical Analysis

Statistical analysis was performed using GraphPad Prism 7 (Graph Pad Software, San Diego, CA, USA). All data were presented as mean ± SD. One-way ANOVA followed by Tukey's post hoc test was used for comparison among multiple groups. Two-way ANOVA was used to analyze the long-term neurobehavioral results. *P* < 0.05 was considered statistically significant.

## 3. Results

### 3.1. Mortality and SAH Grading Score

**Figure 2:**
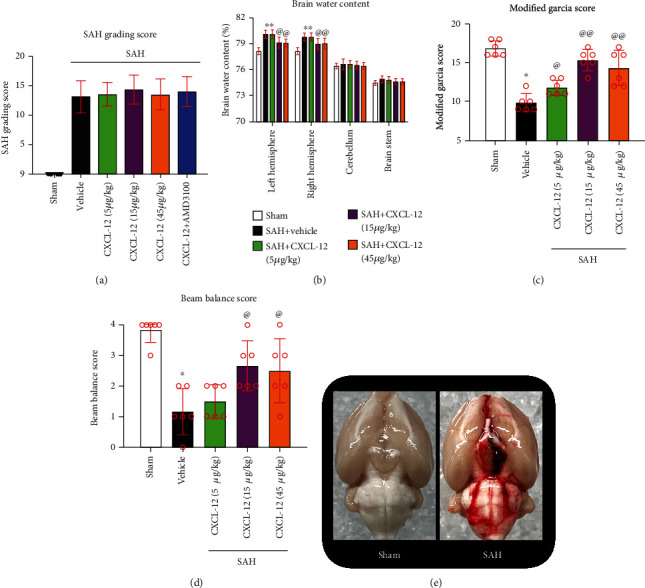
Intranasal administration of rh-CXCL-12 improved short-term neurological outcome and attenuated brain edema at 24 h after SAH. (a) SAH grading score of each group. (b) Quantification of brain water content in the left hemisphere, right hemisphere, cerebellum, and brain stem at 24 h after SAH. (c, d) Intranasal administration of rh-CXCL-12 improved neurological performance on the modified Garcia and beam balance test at 24 h after SAH. (e) Representative pictures of the brain in the sham and SAH groups (subarachnoid blood clots were mainly presented around the circle of Willis). Vehicle: sterile distilled water. Data were presented as mean ± SD. *n* = 6 per group. ^∗^*P* < 0.05 vs. sham group; ^@^*P* < 0.05, ^@@^*P* < 0.01 vs. SAH+vehicle group.

Of the 136 rats used, 112 rats underwent SAH induction. Of which, 15 (14.56%) rats died within 24 h after SAH, and 9 rats were excluded from this project due to mild SAH. There was no mortality in the sham group. Subarachnoid blood clots were distributed around the circle of Willis and ventral brain stem after SAH induction, with a significant difference from the sham group (Figure 2(e)). The average SAH grading scores among all SAH groups showed no significant differences (Figure 2(a)).

### 3.2. Expression Levels of Endogenous CXCL-12, CXCR4, and NLRP1 and Colocalization of CXCR4 with Neurons, Microglia, and Astrocytes after SAH

**Figure 3:**
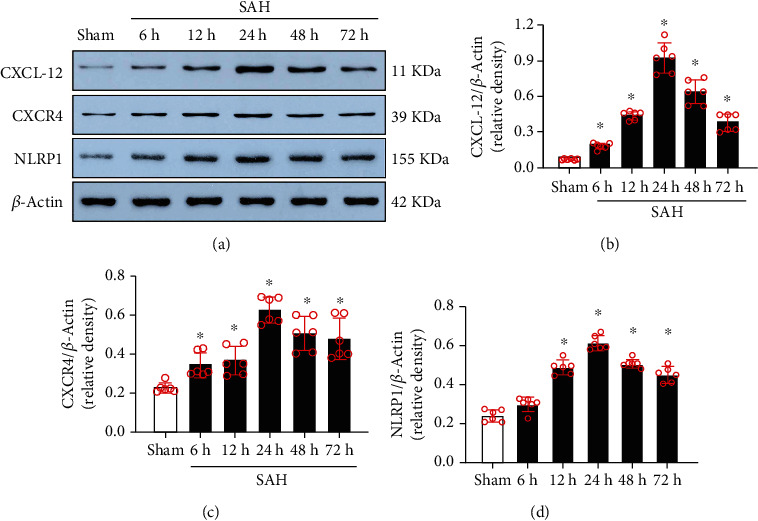
Time course of CXCL-12, CXCR4, and NLRP1, as well as cellular localization of CXCR4 receptor after SAH. (a) Representative western blot bands of time course and densitometric quantification of CXCL-12, CXCR4, and NLRP1 (a–d) in the ipsilateral hemisphere after SAH. Data were presented as mean ± SD. ^∗^*P* < 0.05 vs. sham group; *n* = 6 per group.

**Figure 4:**
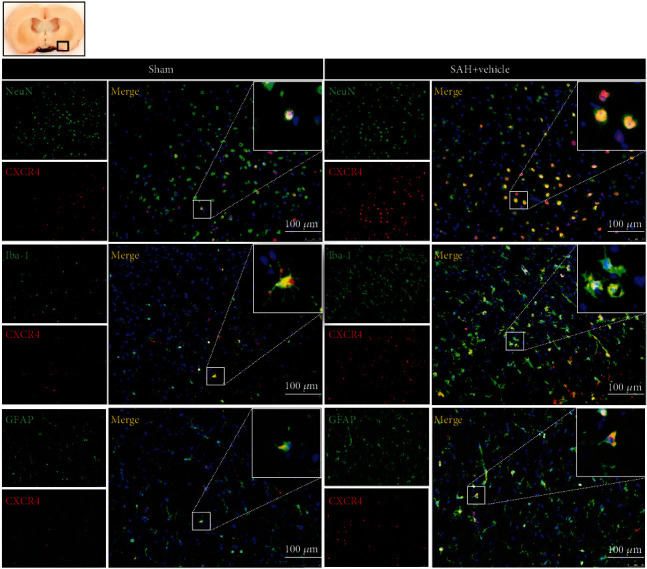
Coimmunofluorescence staining of CXCR4 (red) on neurons (NeuN, green), microglia (Iba-1, green), and astrocytes (GFAP, green) in the ipsilateral basal cortex at 24 h after SAH. Cell nuclei were counterstained with DAPI (blue). A small black square within the coronal section of the brain indicated the location of where the immunofluorescence staining images were taken. *n* = 2 per group. Scale bar = 100 *μ*m.

As shown in Figure 3, the results of Western blotting showed that the endogenous protein expression levels of CXCL-12, CXCR4, and NLRP1 increased in a time-dependent manner, and peaked at 24 h after SAH when compared to the sham group (*P* < 0.05). Coimmunofluorescence staining of Iba-1, NeuN, and GFAP with CXCR4 showed that CXCR4 was expressed on neurons, microglia and astrocytes within cortices in the sham group and in the perihemorrhagic area at 24 h after SAH (showed as Figure 4).

### 3.3. Intranasal Administration of Exogenous rh-CXCL-12 Improved Short-Term Neurobehavioral Dysfunctions and Attenuated Brain Edema at 24 h after SAH

The brain water content in the left and right hemisphere was significantly increased in the SAH+vehicle and SAH+rh-CXCL-12 (5 *μ*g/kg) group, which was significantly reduced by the administration of rh-CXCL-12 at doses of 15 *μ*g/kg and 45 *μ*g/kg (*P* < 0.05, Figure 2(b)). Brain water contents in the cerebellum and brain stem were not significantly different between the sham and SAH groups. The neurobehavioral outcomes of modified Garcia and beam balance were significantly reduced at 24 h after SAH in the SAH+vehicle and SAH+rh-CXCL-12 (5 *μ*g/kg) groups. However, administration of rh-CXCL-12 (15 *μ*g/kg) and rh-CXCL-12 (45 *μ*g/kg) significantly improved the neurological scores at 24 h after SAH (*P* < 0.05, Figures 2(c) and 2(d)). Based on these results, the optimal dose of Rh-CXCL-12 was 15 *μ*g/kg, which was used for the mechanistic studies.

### 3.4. Rh-CXCL-12 Reduced Neuronal Degeneration and Pyroptosis at 24 h after SAH

**Figure 5:**
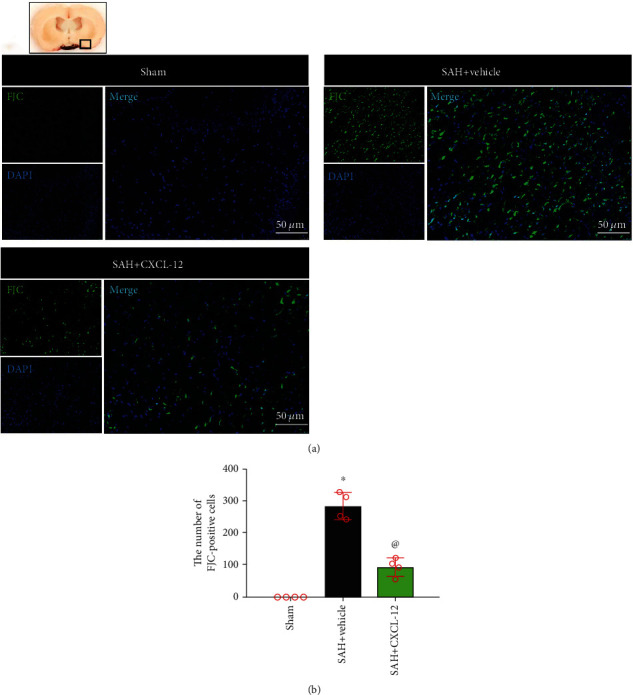
Intranasal administration of rh-CXCL-12 attenuated *neuronal degeneration* at 24 h after SAH. (a) Representative immunofluorescence staining of FJC-positive cells in the ipsilateral basal cortex at 24 h after SAH. Green indicated FJC-positive staining, and blue indicated DAPI-positive nuclear staining. A small black square within the coronal section of the brain indicated the location of where the immunofluorescence staining images were taken. (b) Quantitative analysis of FJC-positive cells. *n* = 4 per group. Vehicle: sterile distilled water. Data were represented as mean ± SD. ^∗^*P* < 0.05 vs. sham group; ^@^*P* < 0.05 vs. SAH+vehicle group.

**Figure 6:**
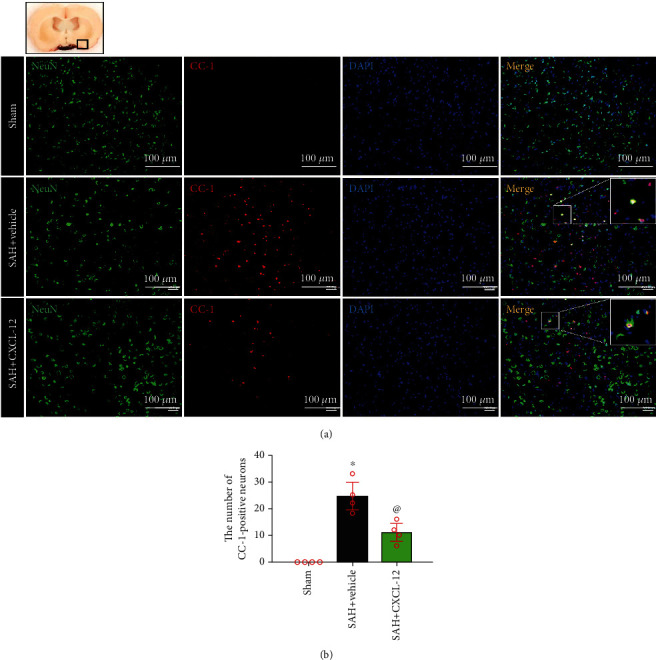
Intranasal administration of rh-CXCL-12 attenuated CC-1-positive neurons after SAH. (a) Representative immunofluorescence staining of CC-1-positive neurons in the ipsilateral basal cortex at 24 h after SAH. Green indicated CC1-positive staining, red indicated neurons, and blue indicated DAPI-positive nuclear staining. A small black square within the coronal section of the brain indicated the location of where the immunofluorescence staining images were taken. (b) Quantitative analysis of CC-1-positive neurons. *n* = 4 per group. Vehicle: sterile distilled water. Data were represented as mean ± SD. ^∗^*P* < 0.05 vs. sham group; ^@^*P* < 0.05 vs. SAH+vehicle group.

**Figure 7:**
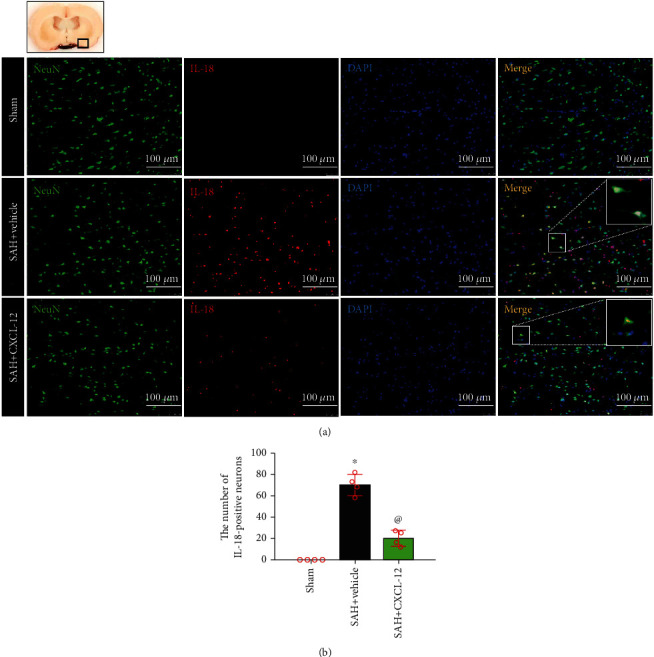
Intranasal administration of rh-CXCL-12 attenuated IL-18-positive neurons after SAH. (a) Representative immunofluorescence staining of IL-18-positive neurons in the ipsilateral basal cortex at 24 h after SAH. Green indicated IL-18-positive staining, red indicated neurons, and blue indicated DAPI-positive nuclear staining. A small black square within the coronal section of the brain indicated the location of where the immunofluorescence staining images were taken. (b) Quantitative analysis of IL-18-positive neurons. *n* = 4 per group. Vehicle: sterile distilled water. Data were represented as mean ± SD. ^∗^*P* < 0.05 vs. sham group; ^@^*P* < 0.05 vs. SAH+vehicle group.

FJC staining showed that the FJC-positive cells were reduced after rh-CXCL-12 administration (Figure 5). Double-immunofluorescence staining of neurons with CC-1 and IL-18 were performed to evaluate neuronal pyroptosis in the ipsilateral basal cortex at 24 h after SAH. Compared with the sham group, rats in the SAH+vehicle group showed an increase in CC-1-positive neurons and IL-18-positive neurons. Rats treated with rh-CXCL-12 had fewer CC-1-positive neurons and IL-18-positive neurons at 24 h after SAH (Figures 6(a) and 7(a)). Quantitative analysis showed that rh-CXCL-12 administration significantly reduced the number of CC-1-positive neurons and IL-18-positive neurons (Figures 6(b) and 7(b)).

### 3.5. Administration of rh-CXCL-12 Attenuated Neuronal Pyroptosis through the CXCR4/NLRP1 Signaling Pathway at 24 h after SAH

**Figure 8:**
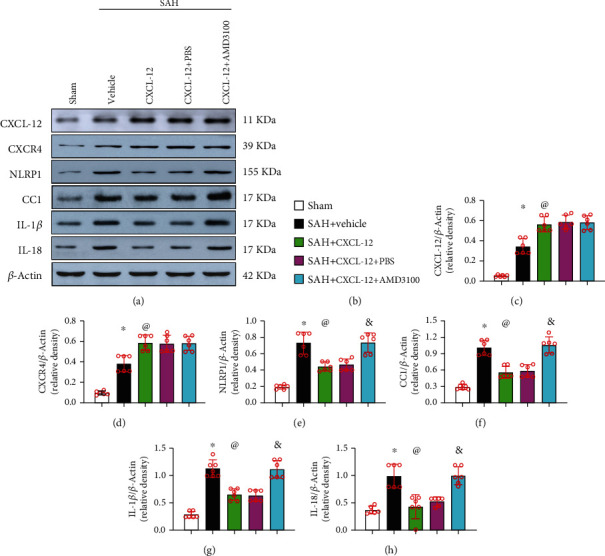
Effects of CXCR4 inhibitor on downstream proteins in the proposed signaling pathway with rh-CXCL-12 treatment at 24 h after SAH. (a) Representative Western blot bands of the proteins CXCL-12, CXCR4, NLRP1, IL-1*β*, CC-1, and IL-18. (b–h) Quantitative analysis of the relative expression level of these proteins at 24 h after SAH. ^∗^*P* < 0.05 vs. sham; ^@^*P* < 0.05 vs. SAH+vehicle group; ^&^*P* < 0.05 vs. SAH+PBS group; *n* = 6 per group.

Western blot results showed that the pathway-related proteins, CXCL-12, CXCR4, NLRP1, IL-1*β*, IL-18, and CC-1, were upregulated in the SAH+vehicle group at 24 h after SAH when compared with the sham group (Figures 8(a)–8(h)). Rh-CXCL-12 treatment further increased the expression levels of CXCL-12 and CXCR4, but decreased the expressions of NLRP1, IL-1*β*, IL-18, and CC-1, compared with the SAH+vehicle group (Figures 8(a)–8(h)). The administration of AMD3100 reversed regulation of pathway-related proteins and the antipyroptosis effects of rh-CXCL-12 at 24 h after SAH (Figures 8(a)–8(h)).

## 4. Discussion

The present study was the first to investigate the neuroprotective effects of CXCL-12 and explore the potential underlying mechanisms after experimental SAH in rats. Our results demonstrated that (1) endogenous protein levels of CXCL-12, CXCR4, and NLRP1 were increased and peaked at 24 h after SAH. The CXCR4 receptors were expressed on microglia, neuron, and astrocytes at 24 h after SAH. (2) Rh-CXCL-12 improved short-term neurological deficits and ameliorated brain edema at 24 h after SAH. Furthermore, rh-CXCL-12 treatment reduced the number of FJC-positive cells, CC-1-positive neurons, and IL-18-positive neurons in the perihemorrhagic area in the ipsilateral cerebral cortex; (3) administration of rh-CXCL-12 significantly increased the expression levels of CXCL-12 and CXCR4, but decreased the expression of NLRP1, IL-1*β*, IL-18, and CC-1; (4) CXCR4 inhibitor, AMD3100, reversed the antipyroptosis effects of rh-CXCL-12 and its effects on the CXCR4/NLRP1 signaling pathway. Taken together, our results showed that the activation of CXCR4 with rh-CXCL-12 may exert a neuroprotective effect and improve neurological function by reducing *neuronal* pyroptosis after SAH, and these effects were at least in part via activation of the CXCR4/NLRP1 signaling pathway.

CXCR4, a chemokine receptor in the G protein-coupled receptor gene family, is widely found in the CNS and immune cells. It induces immune cell migration and nervous system development by binding to its ligand, CXCL-12. CXCR4 is involved in regulating the inflammatory response in CNS diseases, such as Alzheimer's disease [28], ischemic stroke [20], and Parkinson's disease [29]. Furthermore, some other studies have reported that CXCL-12/CXCR4 reduced neuronal apoptosis after traumatic brain injury and ischemic stroke [15, 30]. In the present study, our results showed that the expression of exogenous CXCL-12 and CXCR4 increased at the early stage of SAH and peaked at 24 h after SAH. The increased expression of CXCL-12 and CXCR4 may explain its participation in the endogenous neuroprotective mechanisms after SAH, which were insufficient in overriding the injury. Furthermore, we found that CXCR4 colocalized with neurons, microglia, and astrocytes using the double immunofluorescence method, and the number of CXCR4-positive neurons, microglia, and astrocytes was significantly increased at 24 after SAH, which also indicated that CXCR4 was involved in neuronal pyroptosis in EBI after SAH.

The pathogenesis of SAH is varied, including neuroinflammation, apoptosis, autophagy, and pyroptosis [7, 8, 31, 32]. Musluman et al. investigated the association between apoptotic neurons in the petrosal ganglion 20 days after SAH and found petrosal ganglion ischemia caused neuronal apoptosis [33]. Pyroptosis, different than apoptosis, represents a form of cell death triggered by proinflammatory signal and associated with inflammation [34], which is seen primarily in inflammatory cells such as macrophages and may be triggered by bacterial or pathogen infections. This type of cell death has also been confirmed in traumatic brain injury and acute hemorrhagic stroke [35, 36]. Pyroptosis is activated by two caspase-dependent pathways, including the typical caspase 1 and the atypical caspase 4/5/11 pathway [37]. The cardinal feature of pyroptosis is the requirement for caspase 1 activation. Inflammasome plays an important role in the typical caspase 1 pathway [38, 39]. Caspase 1 is responsible for the maturation of proinflammatory cytokines such as IL-1*β* and IL-18 through inflammasome-dependent pathways [40]. Inflammasomes are multimeric protein complexes; NLR family (NLRP1, NLRP3, NLRC4, NLRP6, and NLRP9) are major inflammasomes. It was shown that the NLRP3 protein is upregulated after SAH and peaked at 24 h, along with the elevation of inflammatory factors, IL-1*β* and IL-18 [41]. The NLRP1 inflammasome, which has been reported to be primarily expressed in neurons and glial cells, is the first member that has been characterized among the NLR family. Among known inflammasomes, NLRP1, NLRP2, NLRP3, and AIM2 were widely studied in CNS disease, especially for NLRP1, which played a key role in SAH via-mediated neuronal pyroptosis [13]. Activation of NLRP1 inflammasome is essential for the regulation of proinflammatory cytokines, and overexpression of inflammatory factors can induce EBI after SAH [13, 42]. Based on this mechanism and targeting, selective pharmacological molecule treatment with inhibition of the NLRP1 inflammasome could decrease neuronal injury after SAH. In the present study, our results showed that the expression of exogenous NLRP1 increased at the early stage of SAH. The increased NLRP1 expression indicated that the inflammasome is involved in the pathogenesis of SAH.

We then evaluated the effects of rh-CXCL-12 in the experimental SAH model. Our results showed that the intranasal administration of rh-CXCL-12, at a dose of 15 *μ*g/kg, reduced brain water content and improved short-time neurobehavioral outcomes, which were used as the best dose of rh-CXCL-12 in further experiment. Meanwhile, we demonstrated strong neuronal degeneration and pyroptosis, as evidenced by increased FJC-positive cells, CC-1-positive neurons, and IL-18-positive neurons at 24 h after SAH, which was consistent with previous investigations [42, 43]. Intranasal administration of rh-CXCL-12 reduced the number of FJC-positive cells, CC-1-positive neurons, and IL-18-positive neurons. The results suggested that treatment of rh-CXCL-12 could attenuate neuronal degeneration and pyroptosis after SAH.

We further investigated the underlying molecular mechanism of CXCL-12-induced antipyroptosis effects after SAH. It has been found that SDF-1 ameliorated NLRP3 inflammasome and pyroptosis in OA synoviocytes by activating AMPK signaling pathway [44]. In this study, we found that rh-CXCL-12 treatment significantly improved the modified Garcia and beam balance scores and increased the expression levels of CXCR4, but decreased the expression of inflammasome NLRP1 and inflammatory cytokines, IL-1*β*, CC-1, and IL-18. Furthermore, CXCR4 inhibitor, AMD3100, reversed the neuroprotective effects of rh-CXCL-12 by upregulating the levels of NLRP1, IL-1*β*, CC-1, and IL-18. Taken together, rh-CXCL-12 attenuated neuronal degeneration and pyroptosis, which functioned, at least in part, by activating the CXCR4/NLRP1 signaling pathway after SAH.

This study has several limitations. First, this study focused primarily on the role of pyroptosis in the EBI phase and assessed the potential mechanisms of pyroptosis at 24 h after SAH. However, pyroptosis in delayed brain injury after SAH should also be investigated in future studies. Second, in addition to pyroptosis, the pathogenesis of SAH includes neuronal apoptosis, oxidative stress, and destruction of the blood-brain barrier; however, the present experiment only focused on pyroptosis. Therefore, the observation and study of other mechanisms should be elucidated in future experiments. Third, CXCR4 has other downstream signaling pathways, such as MEK/ERK and PI3K/Akt [45–47]. Therefore, more experiments are necessary to investigate the possible mechanisms of these signaling pathways and the neuroprotective effects of CXCL-12.

## 5. Conclusions

Our results demonstrated that the activation of CXCR4 with CXCL-12 improved short-term neurological deficits and attenuated neuronal pyroptosis in EBI after SAH in rats. The protective effects of CXCL-12 were at least in part through activation of the CXCR4/NLRP1 signaling pathway. Therefore, early administration of CXCL-12 may provide a therapeutic strategy against brain injury after SAH.

## Data Availability

The data support the findings of this study and are available from the corresponding author upon reasonable request.

## Abbreviations

ANOVA: Analysis of variance
AAALAC: Association for Assessment and Accreditation of Laboratory Animal Care International
BBB: Blood-brain barrier
CNS: Central nervous system
CXCL-12: Cysteine-X-cysteine chemokine ligand 12
CXCR4: CXC, chemokine receptor type 4
EBI: Early brain injury
GFAP: Glial fibrillary acidic protein
i.n: Intranasally
i.p: Intraperitoneal
Iba-1: Ionized calcium-binding adaptor molecule 1
IL-1*β*: Interleukin-1 beta
NeuN: *Neuronal nuclei* antigen
SAH: Subarachnoid hemorrhage
SD: Standard deviation.
