# Correction to “Rh‐CXCL‐12 Attenuates Neuronal Pyroptosis after Subarachnoid Hemorrhage in Rats via Regulating the CXCR4/NLRP1 Pathway”

**DOI:** 10.1155/omcl/9756127

**Published:** 2026-03-25

R. Gu, L. Wang, H. Zhou, X. Wang, C. Lenahan, H. Qu, Y. Liu, S. Li, C. Wei, L. Han, X. Hu, G. Zuo, “Rh‐CXCL‐12 Attenuates Neuronal Pyroptosis after Subarachnoid Hemorrhage in Rats via Regulating the CXCR4/NLRP1 Pathway,” *Oxidative Medicine and Cellular Longevity*, 2021, 6966394, https://doi.org/10.1155/2021/6966394.

In the article, there are errors in Figure 6, introduced by the authors during the figure assembly and highlighted on PubPeer [1]. The correct Figure 6 is shown below:

**Figure 6:**
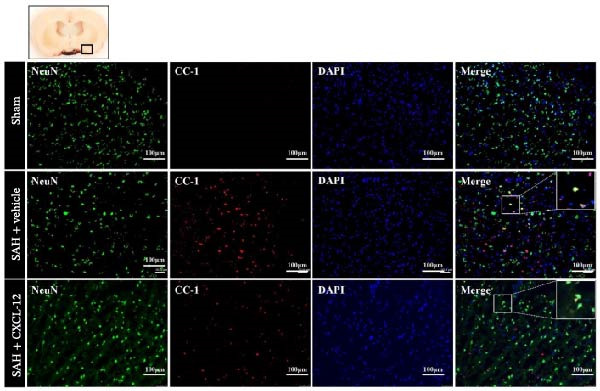
Intranasal administration of rh‐CXCL‐12 attenuated CC‐1‐positive neurons after SAH. (a) Representative immunofluorescence staining of CC‐1‐positive neurons in the ipsilateral basal cortex at 24 h after SAH. Green indicated CC1‐positive staining, red indicated neurons, and blue indicated DAPI‐positive nuclear staining. A small black square within the coronal section of the brain indicated the location of where the immunofluorescence staining images were taken. (b) Quantitative analysis of CC‐1‐positive neurons. *n* = 4 per group. Vehicle: sterile distilled water. Data were represented as mean ± SD.  ^∗^ *P* < 0.05 vs. sham group; ^@^ *P* < 0.05 vs. SAH + vehicle group.

We apologize for these errors.
